# Development and validation of a novel nomogram to predict the risk of the prolonged postoperative length of stay for lumbar spinal stenosis patients

**DOI:** 10.1186/s12891-023-06822-y

**Published:** 2023-09-02

**Authors:** Parhat Yasin, Xiaoyu Cai, Muradil Mardan, Tao Xu, Yakefu Abulizi, Abasi Aimaiti, Huan Yang, Weibin Sheng, Mardan Mamat

**Affiliations:** 1 Department of Spine Surgery, The First Affiliated Hospital of Xinjiang Medical University, Urumqi, 830054 Xinjiang China; 2 Department of Spine center, Xinhua Hospital affiliated to Shanghai Jiaotong University School of Medicine, Shanghai, 200092 China; 3 Department of Anesthesiology, The First Affiliated Hospital of Xinjiang Medical University, Urumqi, 830054 Xinjiang China

**Keywords:** Lumber spinal stenosis, Length of stay, Spine surgery, Risk factors, Nomogram

## Abstract

**Background:**

Lumber spinal stenosis (LSS) is the increasingly reason for spine surgery for elder patients since China is facing the fastest-growing aging population. The aim of this research was to create a model to predict the probabilities of requiring a prolonged postoperative length of stay (PLOS) for lumbar spinal stenosis patients, minimizing the healthcare burden.

**Methods:**

A total of 540 LSS patients were enrolled in this project. The outcome was a prolonged PLOS after spine surgery, defined as hospitalizations ≥ 75th percentile for PLOS, including the day of discharge. The least absolute shrinkage and selection operator (LASSO) was used to identify independent risk variables related to prolonged PLOS. Multivariable logistic regression analysis was utilized to generate a prediction model utilizing the variables employed in the LASSO approach. The receiver operating characteristic (ROC) curve’s area under the curve (AUC) and the calibration curve’s respective curves were used to further validate the model’s calibration with predictability and discriminative capabilities. By using decision curve analysis, the resulting model’s clinical effectiveness was assessed.

**Results:**

Among 540 individuals, 344 had PLOS that was within the usual range of P75 (8 days), according to the interquartile range of PLOS, and 196 had PLOS that was above the normal range of P75 (prolonged PLOS). Four variables were incorporated into the predictive model, named: transfusion, operation duration, blood loss and involved spine segments. A great difference in clinical scores can be found between the two groups (***P*** < 0.001). In the development set, the model’s AUC for predicting prolonged PLOS was 0.812 (95% CI: 0.768–0.859), while in the validation set, it was 0.830 (95% CI: 0.753–0.881). The calibration plots for the probability showed coherence between the expected probability and the actual probability both in the development set and validation set respectively. When intervention was chosen at the potential threshold of 2%, analysis of the decision curve revealed that the model was more clinically effective.

**Conclusions:**

The individualized prediction nomogram incorporating five common clinical features for LSS patients undergoing surgery can be suitably used to smooth early identification and improve screening of patients at higher risk of prolonged PLOS and minimize health care.

## Introduction

Lumbar spinal stenosis (LSS) is considered a common reason for pain in older persons, especially, since China is facing the fastest-growing aging population [1]. Therapy towards LSS can mainly be categorized into two general groups: conservative approaches (e.g., medication, lifestyle interventions, etc.) and surgery treatment (e.g., conventional invasive decompression surgery, minimally invasive decompression surgery, etc.). Surgery would be a better option when conservative treatments cannot still work. More innovation is needed to prevent iatrogenic injury and postoperative morbidity due to rising patient expectations, shorter postoperative length of hospital stays (PLOS), and earlier return to work [2]. However, geriatric patients have more likelihood of being with comorbidities, malnutrition and functional deterioration. Aside from problems linked to the surgical or anesthetic technique, the surgical stress response is a significant determinant in postoperative morbidity [3]. It would trigger the immune and neuro-endocrine system which would make patients experience discomfort, affecting satisfaction [4]. By concentrating on preoperative (such as patient education), intraoperative (surgeons and anesthesiologists), and postoperative (such as early removal of bladder catheter) care, the Enhanced recovery after surgery (ERAS) protocols aim to reduce surgical stress and promote faster postoperative recovery. Consequently, they are beneficial to diminishing morbidity and decreasing the PLOS and costs.

The ERAS program in lumbar spine surgery is receiving more attention because it can decrease postoperative complication rates, postoperative discomfort, and length of stay (LOS) while encouraging physiological function recovery [5–9], since it was first implemented into spine surgery in the mid-2000s [5, 7, 10, 11]. What is more, some studies have revealed the ERAS and uncompromised functional outcomes among elderly patients with reduced PLOS [12, 13]. Lu et al. have developed a visualized tool, i.e., nomogram, using logistic regression concerning lumbar spine fusion surgery [14]. Ken et al. further note that ERAS enhances beneficial recovery of physiologic function and LOS in individuals with frailty following 1–2 levels TLIF [15]. In addition to raising the risk of deep vein thrombosis and hospital-acquired infections, prolonged PLOS is also linked to a number of perioperative poor outcomes and adds to the financial burden on hospitals [4]. However, there was no visualized toolkit for assessing the risk of prolonged PLOS for lumbar spinal stenosis patients.

Therefore, we made the decision to create a practical prediction tool for spine surgeons, nurses, or other healthcare professionals working in the field of spine surgery, to support the identification of patients having the probability of prolonged PLOS. This will improve patient rooms and minimize the healthcare burden. The TRIPOD Checklist is adhered to by the prediction algorithm provided in this study [16].

## Methods

All individual consents for this retrospective study were waived once the research was accepted by our hospital’s ethics committee.

### Patients

Participants in this population-based retrospective cohort research who were deemed to have spinal stenosis and were admitted to the department of spine surgery between January 2019 and December 2022 were recruited with the ethical permission of our institution. All patients who underwent decompression and transforaminal lumbar interbody fusion (TLIF) with pedicle screw instrumentation was enrolled into this study. A posterior midline lumbar incision was made after the patient was positioned prone on a radiolucent table. After the bilateral lamina and facet joints were exposed, the intervertebral disc was exposed and a sufficient amount of posterior decompression was achieved by performing a unilateral facetectomy and a partial laminectomy. After the disc tissue was removed using the reamer, an appropriate-sized cage filled with autologous bone graft was put into the intervertebral gap. In order to reestablish the lordosis and preserve the regained disc height, bilateral pedicle screws and titanium rods were then put in place and axially compressed. The patients in this research concurrently fulfilled the requirements listed below: (1) Diagnosed with spinal stenosis; (2) received surgical treatment; (3) The data was complete and readily available; (4) 18 years or older. The exclusion criteria of alternatives: (1) Patients treated with any minimally invasive spinal surgeries techniques (microscopic / microendoscopic / tabular / mini-open / percutaneous / full-endoscopic based approach, etc.); (2) Revision spinal surgery; (3) Tuberculosis, brucella, pyogenic and unknown spondylitis; (4) Spinal tumor (5) younger 18-year-old; (6) Spinal deformity; (7) Spinal compression fractures; (8) Spinal out of alignment; (9) Patients with missing data were > = 10% excluded from the analysis.

### Collection of data

**Fig. 1:**
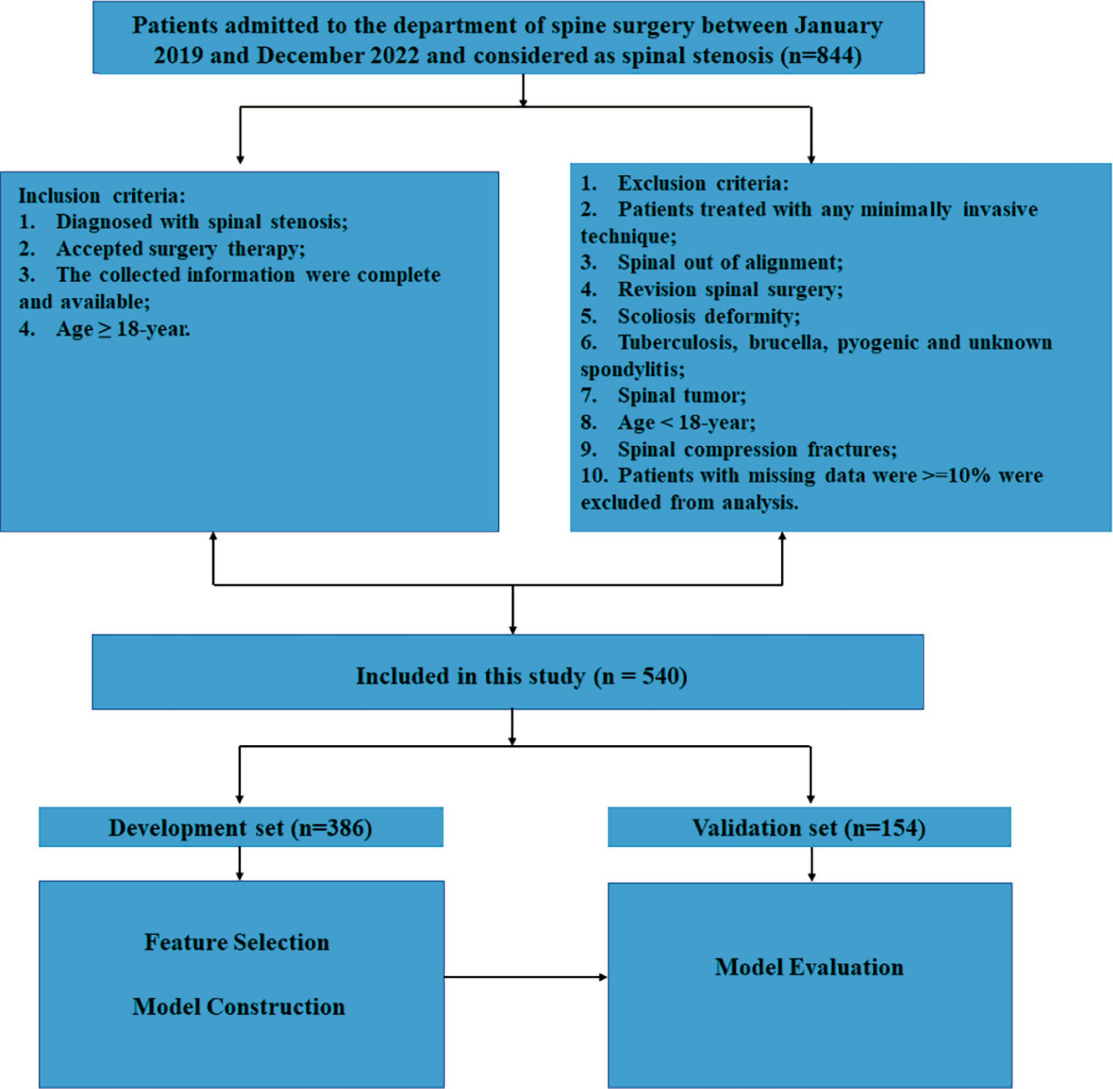
Workflow of this research

Age, gender, ethnicity, body mass index (BMI), and pain intensity were all recorded for each instance. The pain intensity was then classified into two groups based on a visual analog scale (moderate, VAS < 5, and severe, VAS > 5) as well as the patient’s demographic information, symptom durations, affected limb, muscle strength; Pre-operative blood-related indicators gathered like erythrocyte sedimentation rate (ESR), preoperative C-reactive protein (CRP), preoperative white blood cell (WBC), liver and kidney functionality indexes. Past history of patients, in addition to hypertension and diabetes mellitus, contained: cerebrovascular, cardiovascular, hepatic, kidney, thyroid and respiratory diseases. Surgery-associated information, i.e., level of involvement, the day of surgery proceeded, number of the affected vertebras, operation duration, intraoperative infusion volume, blood transfusion and blood loss volume, were collected from medical records. Postoperative length of stay (PLOS) is the total number of days a patient stays in the hospital following surgery before being released. Using the MICE package, we imputed the missing data (version 3.14.0) [17]. We categorized some numeric features to improve the accuracy of our model with its “CUT-OFF” value using pROC package [18]. 70% of the study participants were chosen at random to be the development set, while the other participants were split into the validation set. (Fig. 1)

### Variables selection

Given that many traits have been gathered into this research, it this essential to carry out feature reduction, i.e., variables screening, cause it will lead to overfitting when inputting high-dimensional features [19]. Due to the assignment of the least absolute shrinkage and selection operator (LASSO) to the elimination estimation technique, we find potential predictors using this method [20]. This process is via shrinking some coefficients to zero by penalizing the absolute values of the regression coefficients, which achieves the selection of predictors using the R package “glmnet” [1]. A multivariable logistic regression analysis was then carried out. All variables having a two-sided *P* value 0.05 were included in the model, along with their odds ratios (OR), associated 95% confidence intervals (CI), coefficients, and accompanying p-values.

### Nomogram Construction and Validation

Nomogram was constructed to provide a visualized toolkit for assessing the risk of prolonged PLOS via fitting a multivariate logistic regression consisting of selected features using the rms package. To assess the calibration of this predictive nomogram, calibration curves were produced. The area under the curve (AUC) of the receiver operating characteristics (ROC), which represented the nomogram’s predictive accuracy, was calculated using the “pROC” package to evaluate the model’s discrimination. We used the “rmda” package to perform the Decision curve analysis (DCA) to evaluate the prolonged PLOS risk nomogram’s clinical applicability.

### Statistical analysis

We used R software version 4.1.2 to carry out the statistical analysis. With *Q-Q* plots of all the data, the data’s normality was evaluated. If the continuous variables had a normal distribution, their mean values plus standard deviation (SD) were reported; if not, their median values were (quartile). The Student’s *t*-test was applied to compare two mean values of continuous data that were found to have a normal distribution. *Mann-Whitney U*-test was employed in all other instances. Frequency was used to express categorical variables (percentage). To compare two frequencies, the chi-square test or fisher’s exact test was employed.

**Table 1:** Characteristic at baseline between prolonged PLOS and normal PLOS groups

Characteristics	All (*n* = 540)	Normal (*n* = 344)	Prolonged (*n* = 196)	*P*
**Age (year)**	59.3 ± 13.5	58.3 ± 14.2	61.0 ± 12.1	0.022
**Gender**:				0.201
Female	282 (52.2%)	172 (50.0%)	110 (56.1%)	
Male	258 (47.8%)	172 (50.0%)	86 (43.9%)	
**Symptoms Duration (months)**	38.5 ± 55.6	36.7 ± 57.2	41.9 ± 52.8	0.286
**Affected Limb**:				0.043
Both	196 (36.3%)	112 (32.6%)	84 (42.9%)	
Left	173 (32.0%)	120 (34.9%)	53 (27.0%)	
Right	171 (31.7%)	112 (32.6%)	59 (30.1%)	
**Muscle Strength**:				0.040
3	125 (23.1%)	69 (20.1%)	56 (28.6%)	
4	346 (64.1%)	225 (65.4%)	121 (61.7%)	
5	69 (12.8%)	50 (14.5%)	19 (9.69%)	
**Pain Degree**:				1.000
Moderate	343 (63.5%)	219 (63.7%)	124 (63.3%)	
Severe	197 (36.5%)	125 (36.3%)	72 (36.7%)	
**Hypertension**:				0.411
No	295 (54.6%)	193 (56.1%)	102 (52.0%)	
Yes	245 (45.4%)	151 (43.9%)	94 (48.0%)	
**DM**:				0.366
No	410 (75.9%)	266 (77.3%)	144 (73.5%)	
Yes	130 (24.1%)	78 (22.7%)	52 (26.5%)	
**Cardiovascular Diseases**:				0.140
No	461 (85.4%)	300 (87.2%)	161 (82.1%)	
Yes	79 (14.6%)	44 (12.8%)	35 (17.9%)	
**Cerebrovascular Diseases**:				0.015
No	498 (92.2%)	325 (94.5%)	173 (88.3%)	
Yes	42 (7.78%)	19 (5.52%)	23 (11.7%)	
**Hepatic Diseases**:				0.289
No	480 (88.9%)	310 (90.1%)	170 (86.7%)	
Yes	60 (11.1%)	34 (9.88%)	26 (13.3%)	
**Respiratory Diseases**:				0.346
No	507 (93.9%)	326 (94.8%)	181 (92.3%)	
Yes	33 (6.11%)	18 (5.23%)	15 (7.65%)	
**Previous Surgery**:				0.175
No	314 (58.1%)	208 (60.5%)	106 (54.1%)	
Yes	226 (41.9%)	136 (39.5%)	90 (45.9%)	
**Kidney Diseases**:				0.072
No	510 (94.4%)	330 (95.9%)	180 (91.8%)	
Yes	30 (5.56%)	14 (4.07%)	16 (8.16%)	
**BMI** (Kg/m^2^)	25.4 ± 3.70	25.2 ± 3.67	25.8 ± 3.74	0.066
**Smoker**:				1.000
No	451 (83.5%)	287 (83.4%)	164 (83.7%)	
Yes	89 (16.5%)	57 (16.6%)	32 (16.3%)	
**Alcohol Abuses**:				0.531
No	463 (85.7%)	292 (84.9%)	171 (87.2%)	
Yes	77 (14.3%)	52 (15.1%)	25 (12.8%)	
**WBC** (10^9^/L)	6.63 ± 2.13	6.52 ± 1.97	6.80 ± 2.37	0.161
**HB** (g/L)	138.0 ± 16.2	138.0 ± 15.8	136.0 ± 16.7	0.102
**Platelet** (10^9^/L)	237.0 ± 66.7	235.0 ± 68.6	242.0 ± 63.1	0.255
**ESR** (mm/h)	20.3 ± 15.3	19.4 ± 14.9	22.0 ± 15.8	0.067
**CRP** (mg/L)	6.93 ± 15.2	6.71 ± 15.7	7.31 ± 14.5	0.651
** K** (mmol/L)	3.86 ± 0.35	3.86 ± 0.34	3.88 ± 0.37	0.547
**Na** (mmol/L)	141.0 ± 6.03	141 ± 7.28	141 ± 2.70	0.841
**Creatinine** (µmol/L)	67.4 ± 28.0	67.5 ± 17.3	67.4 ± 40.5	0.965
**eGFR** (mL/min/1.73 m^2^)	94.0 ± 18.0	94.5 ± 17.6	93.2 ± 18.7	0.419
**ALB** (g/L)	42.2 ± 3.92	42.6 ± 3.63	41.7 ± 4.34	0.016
**AST** (U/L)	20.6 ± 10.3	20.8 ± 11.2	20.3 ± 8.43	0.580
**ALT** (U/L)	23.8 ± 19.6	23.9 ± 21.2	23.6 ± 16.4	0.861
**Day**:				0.413
Friday	70 (13.0%)	43 (12.5%)	27 (13.8%)	
Monday	100 (18.5%)	54 (15.7%)	46 (23.5%)	
Saturday	18 (3.33%)	11 (3.20%)	7 (3.57%)	
Sunday	10 (1.85%)	5 (1.45%)	5 (2.55%)	
Thursday	115 (21.3%)	86 (25.0%)	29 (14.8%)	
Tuesday	122 (22.6%)	75 (21.8%)	47 (24.0%)	
Wednesday	105 (19.4%)	70 (20.3%)	35 (17.9%)	
**Postoperative drainage first Day (ml)**	133 ± 163	135 ± 173	128 ± 146	0.612
**Operation Duration (min)**:				< 0.001
< 150	202 (37.4%)	175 (50.9%)	27 (13.8%)	
>= 150	338 (62.6%)	169 (49.1%)	169 (86.2%)	
**Infusion Volume (mL)**	1493 ± 538	1407 ± 497	1644 ± 573	< 0.001
**Blood Loss (mL)**:				< 0.001
< 200	243 (45.0%)	204 (59.3%)	39 (19.9%)	
>= 200	297 (55.0%)	140 (40.7%)	157 (80.1%)	
**Segment (s)**:				< 0.001
1	313 (58.0%)	226 (65.7%)	87 (44.4%)	
2	158 (29.3%)	109 (31.7%)	49 (25.0%)	
3	66 (12.2%)	9 (2.62%)	57 (29.1%)	
4	3 (0.56%)	0 (0.00%)	3 (1.53%)	
**Transfusions**:				< 0.001
No	470 (87.0%)	323 (93.9%)	147 (75.0%)	
Yes	70 (13.0%)	21 (6.10%)	49 (25.0%)	

DM: Diabetes mellitus, BMI: Body mass index (BMI, Kg/m2),WBC: preoperative white blood cell (WBC, ×109/L), ESR: preoperative erythrocyte sedimentation rate (ESR, mm/h), CRP: preoperative C-reactive protein (CRP, mg/L), Hb: preoperative hemoglobin (Hb, g/L), estimated glomerular filtration rate (eGFR, ml/min/1.73 m2) ALB: pre-operative operative albumin (ALB, g/L), AST: preoperative aspartate aminotransferase (AST, U/L ), ALT: preoperative alanine aminotransferase (ALT, U/L).

## Results

### Patients

The interquartile range of PLOS in 540 patients showed that 344 patients had PLOS of P75 (8 days), which is considered to be a normal PLOS, whereas 196 patients had PLOS of > P75, which is considered to be a protracted PLOS (Fig. 1). The two groups’ perioperative clinical features and complete patient data, including demographics, are shown in Table 1. Between the two groups, statistically significant variations were detected, in infusion volume (*P* < 0.001), operation duration (*P* < 0.001), affected segments number (*P* < 0.001) and transfusion (*P* < 0.001). Compared to patients with normal PLOS, patients with prolonged PLOS were more likely to present elder age (*P* = 0.022), higher ESR (*P* = 0.067) and lower ALB (*P* = 0.016). There was a significant difference discovered between the two groups considering gender, symptom duration and most of the past history before their admission.

### Feature selection

**Fig. 2:**
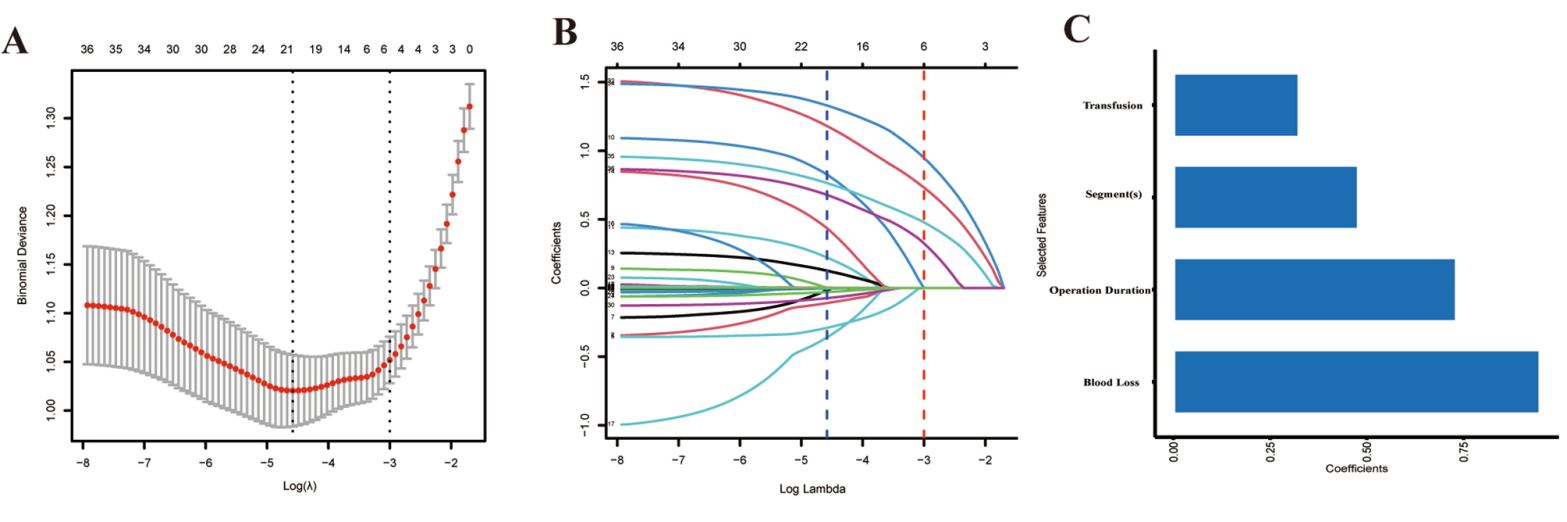
Feature selection. **(A)** The left dotted vertical line represents the minimal criterion, and the 1-SE of the minimum criteria is used to determine the best parameter (lambda) selection in the LASSO model (the right dotted vertical line). **(B)** profiles of the 36 features’ LASSO coefficients. **(C)** Feature selected using LASSO and its coefficients. LASSO, least absolute shrinkage and selection operator;

We screened four nonzero coefficients from thirty-six variables via the LASSO method with the 1-SE of the minimum criteria. (Figure 2A and B C). These variables included operation duration (OR, 3.01; 95% CI, 1.85–5.03; *P* < 0.001), blood loss (OR, 3.57; 95% CI, 2.46–5.76; *P* < 0.001), involved spine segment(s) (OR, 7.99; 95% CI, 3.79–18.6; *P* < 0.001), transfusion (OR, 2.84; 95% CI, 1.48–5.58; *P* = 0.002). (Table 2). Considering that previous studies have found that some clinical characteristics are the risk factors of prolonged PLOS, we have adjusted our model by introducing other features and comparing their discrimination ability. (Table 3) We maintain that there is no obvious improvement after adding these predictors and this will increase the complexity of our model which will decrease the practicality.

**Table 2:** Selected Features for PLOS after surgery

Characteristics	OR^1^	95% CI^1^	*P*
**Operation Duration (min):**			
< 150	—	—	
>= 150	3.01	1.85, 5.03	< 0.001
**Blood Loss (ml)**:			
< 200	—	—	
>= 200	3.57	2.24, 5.76	< 0.001
**Segment (s)**:			
1	—	—	
2	1.19	0.71, 1.97	0.5
3	7.99	3.79, 18.6	< 0.001
4	1,350,582	0.00, NA	> 0.9
**Transfusion**:			
No	—	—	
Yes	2.84	1.48, 5.58	0.002

^1^OR = Odds Ratio, CI = Confidence Interval

**Table 3:** Different model performance

Models	Auc.	Sen.	Spe.	Acc.	Npv.	Ppv.	Rec.	P*
M1^a^	0.812	0.519	0.949	0.801	0.789	0.841	0.519	
M2^a^	0.823	0.519	0.949	0.801	0.789	0.841	0.519	
M3^a^	0.818	0.556	0.925	0.798	0.799	0.796	0.556	
M1^a^ vs. M2^a^								0.892
M1^a^ vs. M3^a^								0.168
M2^a^ vs. M3^a^								0.244
M1^b^	0.826	0.571	0.945	0.792	0.761	0.878	0.571	
M2^b^	0.822	0.571	0.945	0.792	0.761	0.878	0.571	
M3^b^	0.824	0.556	0.912	0.766	0.748	0.814	0.556	
M1^b^ vs. M2^b^								0.641
M1^b^ vs. M3^b^								0.719
M2^b^ vs. M3^b^								0.577

Auc., area under the curve; Sen., sensitivity; Spe., specificity; Acc., accuracy; NPV., negative predictive value; P.P.V., positive predictive value

M1a, M2a, M3a are the models that are fitted using developing dataset; M1b, M2b, M3b are the models that are fitted using validation dataset. *P means the Delong test that compare the AUC value of different models. M1 represents the find model; M2 adjusted infusion volume, age; M3 adjusted albumin

### Clinical score and model performance validation

**Fig. 3:**
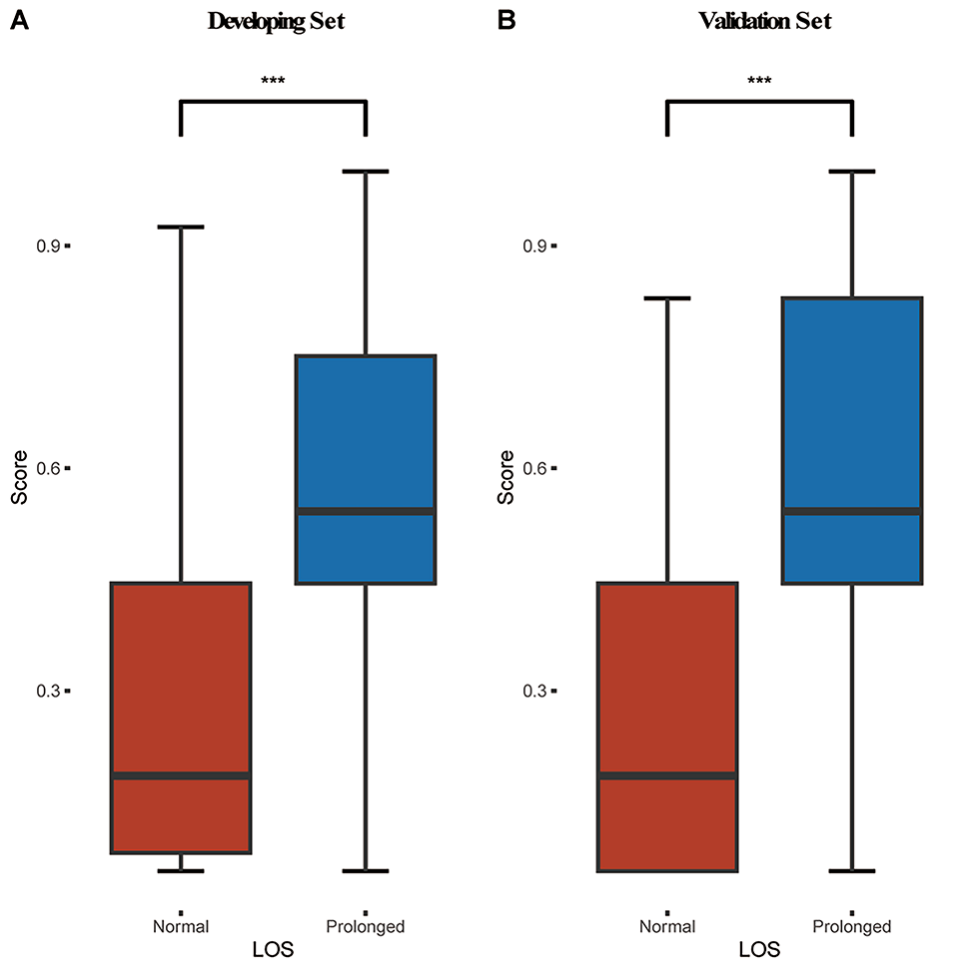
Model Score comparison **(A)** Development set. **(B)** Validation Set. P values were calculated via two independent samples *t*-tests

**Fig. 4:**
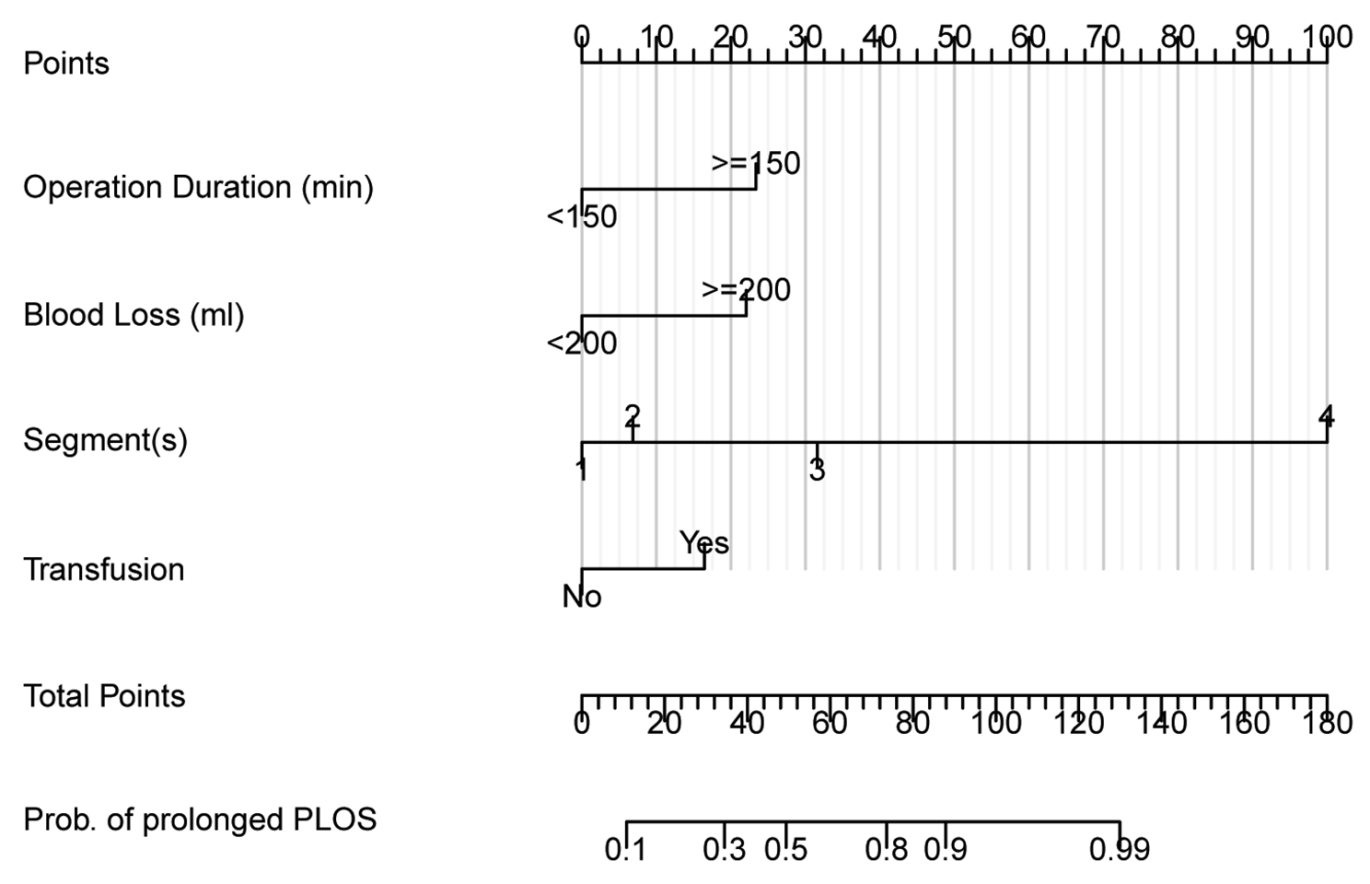
A nomogram predicting the risk of prolonged postoperative length of stay for lumbar spinal stenosis patients Each variable’s value received a score on the point scale axis. Each individual score may be added together to create a total score. By projecting the entire score to the lower total point scale, we can determine the likelihood of a prolonged postoperative length of stay. Typical case: operation duration: 160 min = 22.5, blood loss: 250 ml = 22.5, segments 3 = 32.5 transfusion: no = 0, calculating the sum of above scores reaching a total point of 77.5 which showing that the possibility of prolonged PLOS was higher than 80%

We constructed clinical scores using the multivariate model established above. A great difference can be found between prolonged PLOS and normal PLOS in the development set and validation set respectively. (Fig. 3) Next, to individualize this scoring system, we build a nomogram with the aforementioned model (Fig. 4).

**Fig. 5:**
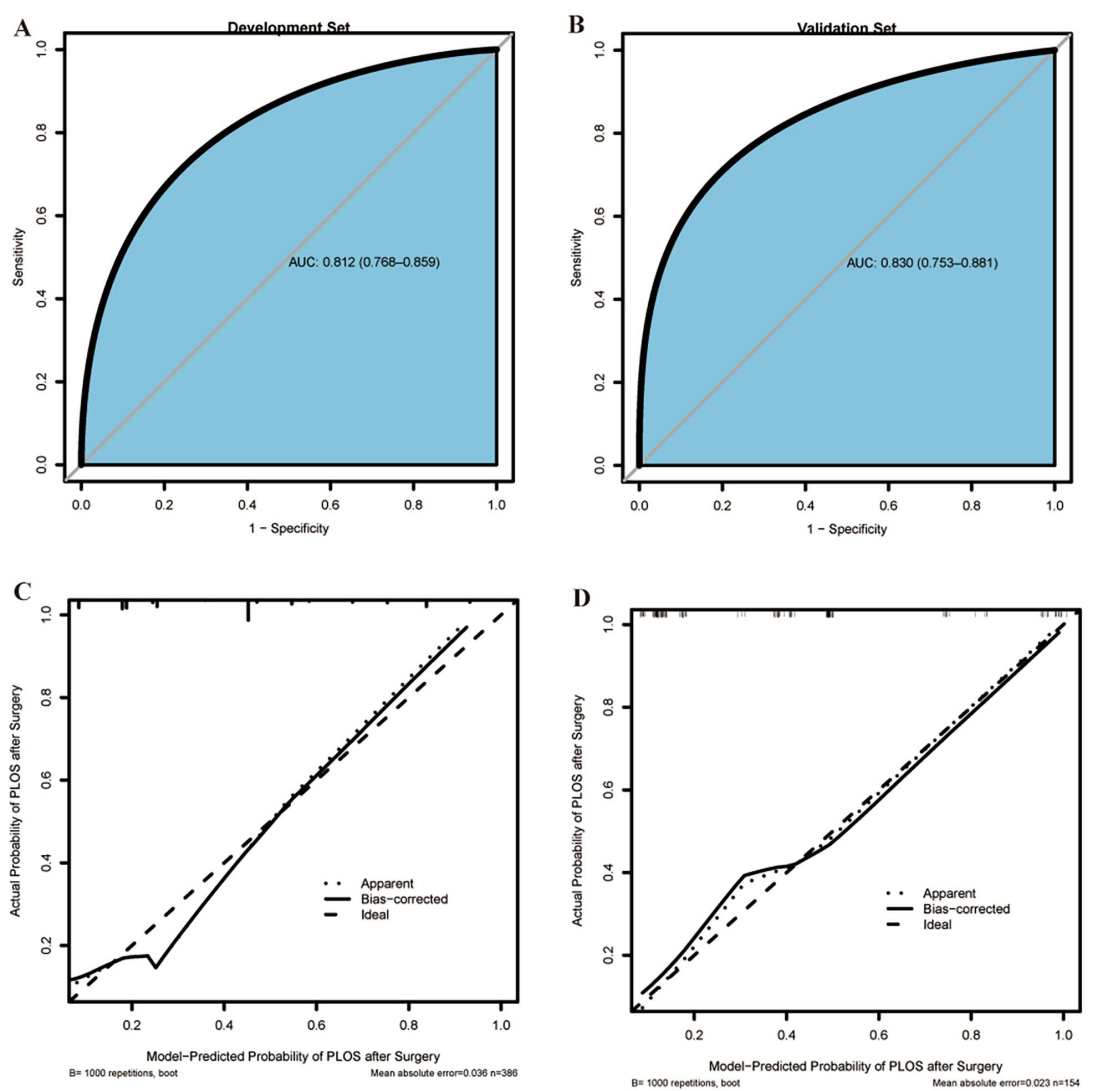
The ROC curves and calibration curves. Receiver-operating characteristic (ROC) curves and calibration curves of the model. **(A)** Development set (ROC). **(B)** Validation set (ROC). **(C)** Development set (Calibration curves). **(D)** Validation set (Calibration curves)

**Fig. 6:**
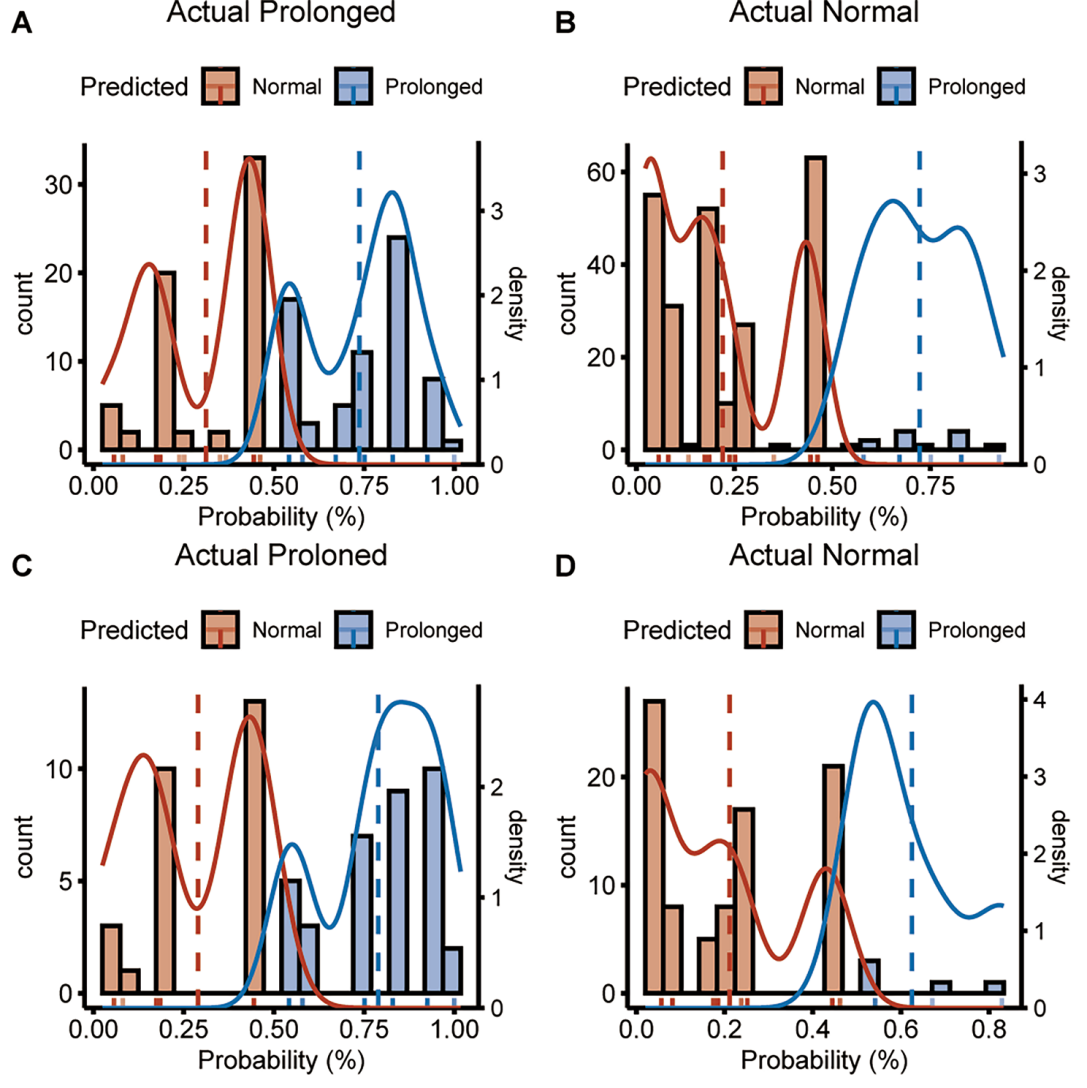
Patients probability density distribution. **(A)** The actual observed prolonged PLOS in development set. **(B)** The actual observed normal PLOS in development set. **(C)** The actual observed prolonged PLOS in validation set. **(D)** The actual observed normal PLOS in validation set

The AUC associated with the prolonged PLOS nomogram in the development set was 0.812 (95% CI, 0 0.768–0.859) and was confirmed to be 0.830 (95% CI, 0.753–0.881) in the validation set (Fig. 5a and b), showing the predictive model has better discrimination. The calibration plot of the predictive model indicated strong concordance performance between the prediction and observation in the development set and validation set respectively (Fig. 5c and d**)** which showed that there is no significant deviation between predicted and actual probability in both sets. Then, we investigated the probability density plot to reveal the probability distribution between the actual observed and predicted (Fig. 6).

### Clinical efficiency of the model

**Fig. 7:**
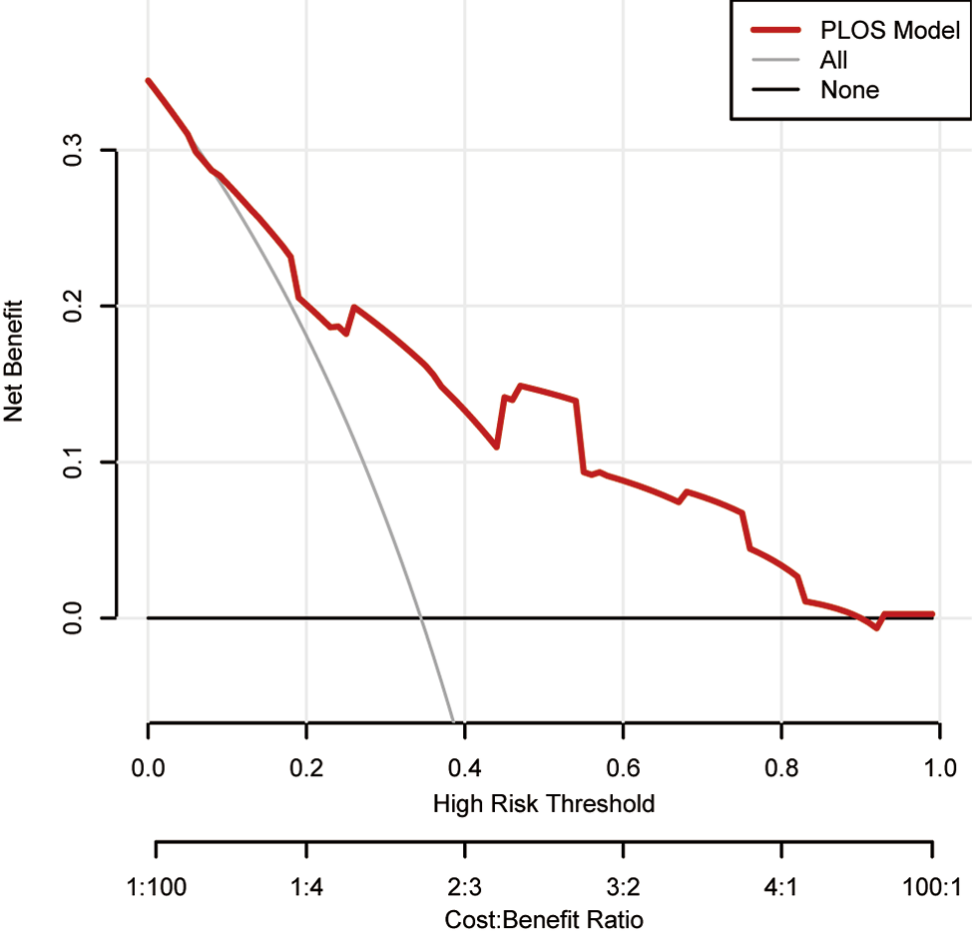
Analysis of the decision curve for the prolonged PLOS prediction model

To determine whether DCA could be advantageous in clinical practice, we put it to use. By assisting in the early identification of individuals at risk for extended PLOS, the DCA showed that this model significantly improved patient outcomes when compared to either treat-all or treat-none strategies. Figure 7 demonstrates that the model had a notable capacity to enhance clinical effectiveness in predicting extended PLOS when threshold probabilities are higher than 2%.

## Discussion

There is now more emphasis on adopting cost-effective strategies that produce the greatest clinical results as a result of payors’ incorporation of quality criteria in their reimbursement choices as a result of rising healthcare expenses. People in poor nations and regions are experiencing increasing worries and uncertainties, particularly in light of the coronavirus illness (COVID-19) outbreak on the global public health scene. Because the pandemic may make poverty’s unequal burden worse [21]. Nevertheless, LOS is tightly conned with costs.

In this research, single-center analysis, we demonstrate that the predictive model by logistic regression is a useful toolkit with a rational basis for selecting 8 days as a “cut-off” for defining prolonged PLOS, evaluating the factors affecting PLOS and developing a model to predict the probability of prolonged PLOS in LSS patients. Features selected after the LASSO method, where unimportant features are filtered by penalizing their coefficients to zero, are operation duration, intraoperative blood loss, the number of affected spinal segments and whether patients had received a transfusion. Based on these four parameters, we have built a model to predict and the result revealed our model has better prediction ability (AUC: 0.812 (95%CI: 0.768–0.859) in development set and 0.830 (95%CI: 0.753–0.881) validation set). Furthermore, we compared the score calculated by our model in both the development and validation set, where we can find significant differences between normal and prolonged PLOS patients.

The use of nomograms in clinical practice is a helpful complement in conversations with patients [22, 23]. As a convenient model-visualizing tool, it can well display the multivariate logistic regression model, where every risk factor was allocated a score depending on its influence on the outcome and can be calculated bedside in clinical daily practice. Previous researchers have used this toolkit in their own field [24, 25]. Lu et al. [14] enrolled 310 patients who underwent open lumber fusion surgery and analyzed prolonged PLOS risk factors and also visualized the model as a nomogram. To relieve pain and nerve compression, patients with degenerative disc disease, lumber canal stenosis, spondylolisthesis, scoliosis failed back surgery, and traumas can benefit from lumber fusion surgery, which includes posterior lumber fusion (PLF), posterior lumber interbody fusion (PLIF), and transforaminal lumber interbody fusion (TLIF). In this study, we specified our target population-spinal stenosis patients with no limits on affected segment numbers. Thus, we believe our model can be more pertinent.

The clinical symptom combination known as lumbar spinal stenosis involves low back pain, discomfort in both lower extremities, paresthesia, and other neurologic abnormalities. Epidural fibrosis, sacroiliac joint pain, disc herniation, facet joint pain, and improper surgery have all been proposed as secondary reasons for the unrelenting pain and incapacity in the low back and lower limbs after lumbar spine surgery [26] However, in our results, the pain degree which is quantified by visual analogue scales (VAS) first and categorized is not related to prolonged PLOS. Yet, the mechanism behind lower back pain is still needed to be explored [27–29]. The mean age is significantly different between the two groups, which is in line with previous studies [30, 31]. Nevertheless, age was not selected when screening the features. Thus, it was not imported into our final model and we did still get discrimination performance. In addition, our results demonstrated that loss of muscle strength is connected to prolong PLOS. A similar pattern of results was obtained in the intensive care unit (ICU) patients, where the loss of ventilation muscle strength will increase the risk of increased length of stay [32, 33]. But the potential mechanism of this on LSS patients is unclear, which can be investigated further.

The time spent in and out of the operating room is known as the operational duration [34]. Wang et al. [25] revealed operation duration is a risk factor for total hip arthroplasty. However, Basques et al. [35] found that operation duration is not significantly related to postoperative LOS. We believe the duration of open surgery will increase the risk of prolonged PLOS for LSS patients as this provided more challenges for postoperative rehabilitation (pain management, removal of urinary catheter, etc.) and lengthier analgesic operation times, which might raise the risk of problems.

According to earlier studies, intraoperative blood loss was one of the surgically associated risk factors for a protracted hospital stay [36–38]. In this research, we found our intraoperative blood loss was categorized with 200 ml being a reference. Blood loss during lumbar spinal fusion surgery often results from intramedullary hemorrhage and bleeding at the surgical site. Li et al. reported the estimated blood loss in multilevel spinal fusion operation can be varied from 750 to 1500 ml [39]. Excessive blood loss in surgery will reduce organ perfusion, which would impact the oxygenation of tissues. Henry et al. [40] pointed out that hypoperfusion after abdominal surgery will increase the morbidity and mortality rate. Thus, it is obvious that blood loss will influence the procedure of postoperative rehabilitation.

In order to prevent morbidities such as hypotension and hypoperfusion with organ impairment, as well as coagulopathy, excessive blood loss may necessitate an allogenic blood transfusion. Many studies have outlined the surgery-related variables like operating duration, and multilevel operations that are linked to the requirement for blood transfusions in spine surgery [41–43]. However, in addition to having a variable cost per unit transfused, blood transfusion has been documented to be linked to a greater risk of developing wound infections, pulmonary embolism, acute lung injury and prolonged hospital length of stay (LOS) [44, 45]. Neef et al. [46] indicated that transfusion was a risk factor after elective primary meningioma resection, which was consistent with our result. The transfusion rate can be ranged from 20.0 to 35% in previous studies [47–49]. In our study, we found the incidence rate of transfusion in fusion surgery was 13%. In short, the indication of transfusion should be strictly followed. Surgery can reduce blood loss by using high infusion volumes, which increase hemodynamic stability. Infusion volume in our study is also meaningfully unalike between the two groups. These results tie well with other studies about rectal surgery and ICU hospitalization [50, 51]. Considering the multicollinearity of infusion volume with blood loss, we did not introduce this variable, which was also filtered through LASSO.

## Limitations

This study has several limitations. First, although internal and self-verification validation was performed for this study, external validation was not. Second, in this series, it was challenging to pinpoint the illness phase. However, the latter is also crucial to demonstrate both the model’s lack of accuracy and its precision. Third, even though the model’s internal validation produced excellent calibration and optimal discrimination, this nomogram still needs external validation using additional databases.

## Conclusions

This model demonstrates effectiveness in the early identification of patients who may experience extended PLOS and in giving a relevant reference for clinical decision-making, which will be useful for coordinating medical resources. In addition, we created and tested a nomogram that may be used to forecast how long PLOS will last for LSS patients.

## Data Availability

The raw data of this article will be made available by the corresponding authors on reasonable requests without any reservation.
